# Construction of a prognostic model and identification of key genes in liver hepatocellular carcinoma based on multi-omics data

**DOI:** 10.1038/s41598-025-98038-4

**Published:** 2025-04-18

**Authors:** Kun Tang, Mingjiang Liu, Cuisheng Zhang

**Affiliations:** Department of Hepatobiliary Surgery, The Affiliated Yantai Yuhuangding Hospital of Qingdao University, Yantai, Shandong Province China

**Keywords:** Liver hepatocellular carcinoma, Immunogenic cell death, Cellular senescence, Risk score, Liver cancer, Cancer genomics

## Abstract

Liver hepatocellular carcinoma (LIHC) strongly contributes to global cancer mortality, highlighting the need for a deeper understanding of its molecular mechanisms to enhance patient prognosis and treatment approaches. We aimed to investigate the differential expression of immunogenic cell death-related genes (ICDRGs) and cellular senescence-related genes (CSRGs) in LIHC and their effects on patient prognosis. We combined the GSE25097, GSE46408, and GSE121248 datasets by eliminating batch effects and standardizing the data. After processing, 16 genes were identified as ICDR&CSR differentially expressed genes (ICDR&CSRDEGs), including *UBE2T*, * HJURP*, * PTTG1*, * CENPA*, and *FOXM1*. Gene set enrichment analysis indicated a strong enrichment of these genes in pre-Notch expression and processing. Gene set variation analysis revealed 20 pathways with significant differences between the LIHC and control groups. Mutation analysis identified *TP53* as the most commonly mutated gene in LIHC samples. A prognostic risk model integrating 12 ICDR&CSRDEGs was developed, showing high precision at 1 year but diminished accuracy at 2 and 3 years. Our constructed prognostic risk model provides valuable insights for predicting patient outcomes and may guide future therapeutic interventions targeting these specific genes. Further research is needed to explore the mechanistic roles of these genes in LIHC progression and treatment response.

## Introduction

Liver hepatocellular carcinoma (LIHC) is a prevalent cancer with high incidence and mortality worldwide. LIHC constitutes more than 80% of liver cancers, ranking among the top three in 46 countries and top five in 90 countries^1^. The limited accuracy of biomarkers for early diagnosis and prognosis often leads to the detection of most LIHC cases at advanced stages, restricting effective treatment options and resulting in high mortality rates. The shift in epidemiological trends among at-risk patients, notably the increase in non-viral causes, has significantly affected primary prevention, monitoring, and treatment strategies^2^. Consequently, identifying new biomarkers and therapeutic targets is crucial to improve the prognosis in patients with LIHC.

Immunogenic cell death (ICD) and cellular senescence are critical biological processes that have garnered much attention in cancer research. ICD is a regulated cell death process that triggers an immune response to dead cell antigens, which is crucial for antitumor immunity^3^. Cellular senescence, marked by permanent cell cycle arrest, initially suppresses tumorigenesis but may later promote tumor progression through a senescence-associated secretory phenotype^4^. Recent research underscores the pivotal roles of ICD and cellular senescence in tumor progression and treatment responses across multiple cancers, such as liver, colorectal, breast, lung, ovarian, and prostate cancers and melanoma^5–7^.

ICD is remarkable in the context of cancer treatment because it improves the effectiveness of chemotherapy in colorectal cancer by facilitating the recruitment and activation of dendritic cells and cytotoxic T lymphocytes^8^. Cellular senescence is linked to chemotherapy resistance in breast cancer cells, indicating that targeting senescence pathways may enhance treatment efficacy^9^. These findings underscore the potential of ICD and cellular senescence as therapeutic targets in cancer treatment. However, the role of immunogenic cell death-related genes (ICDRGs) and cellular senescence-related genes (CSRGs) in LIHC remains largely unexplored. Given the aggressive nature of LIHC and limited effectiveness of current therapies, investigating the differential expression of ICDRGs and CSRGs in LIHC may provide valuable insights into novel prognostic biomarkers and therapeutic targets.

In this study, we aimed to investigate the differential expression of ICDRGs and CSRGs in LIHC and their effects on patient prognosis. To this end, we integrated data from three Gene Expression Omnibus datasets (GSE25097, GSE46408, and GSE121248) with TCGA-LIHC dataset from The Cancer Genome Atlas (TCGA). We utilized DESeq2 to identify differentially expressed genes (DEGs) and intersected these with ICDRGs and CSRGs to derive genes associated with ICD and cellular senescence (ICDR&CSRGs). We performed functional enrichment analyses using gene ontology (GO), Kyoto Encyclopedia of Genes and Genomes (KEGG), gene set enrichment analysis (GSEA), and gene set variation analysis (GSVA). A prognostic risk model was developed using Cox regression analysis. Single-sample GSEA (ssGSEA) was employed to evaluate immune-cell infiltration, and the prognostic model underwent validation through multiple statistical techniques.

Our research revealed significant expression of ICDR&CSRDEGs in LIHC, with potential implications for patient prognosis. Functional enrichment analyses identified the involvement of these genes in key biological processes and pathways. The prognostic risk model demonstrated robust predictive accuracy, and the immune-cell infiltration analysis provided insights into the tumor immune microenvironment. The results underscore the importance of ICD and cellular senescence in LIHC and suggest that targeting these pathways could improve therapeutic outcomes for patients with LIHC.

## Results

### Technology roadmap

**Fig. 1:**
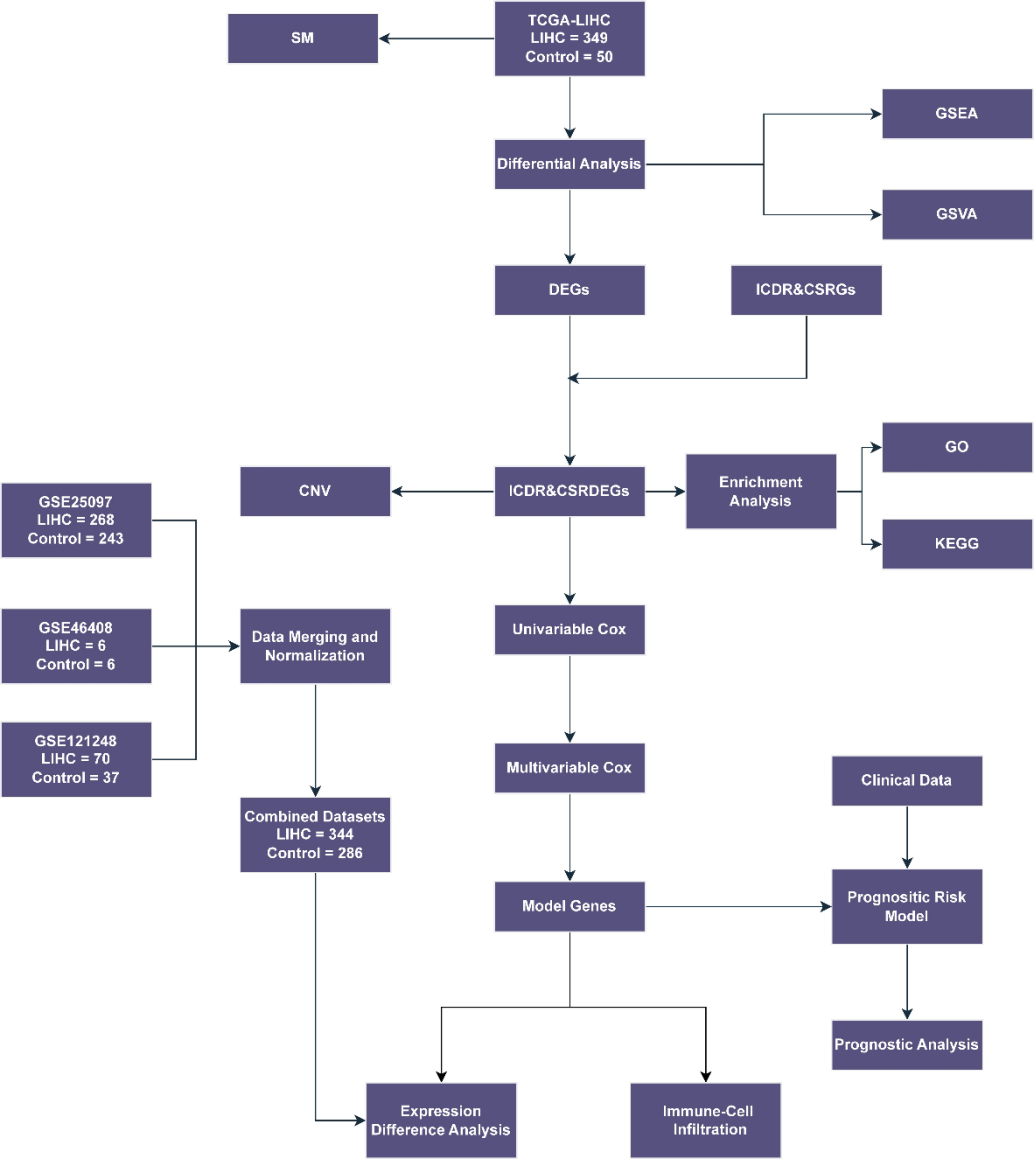
Flow chart showing the comprehensive analysis of ICDR&CSRDEGs. TCGA, The Cancer Genome Atlas; LIHC, Liver Hepatocellular Carcinoma; DEGs, Differentially Expressed Genes; ICDR&CSRGs, Immunogenic Cell Death-Related and Cellular Senescence-Related Genes; ICDR&CSRDEGs, Immunogenic Cell Death-Related and Cellular Senescence-Related Differentially Expressed Genes; SM, Somatic Mutation; CNV, Copy Number Variation; GSEA, Gene Set Enrichment Analysis; GSVA, Gene Set Variation Analysis; GO, Gene Ontology; KEGG, Kyoto Encyclopedia of Genes and Genomes.

A flow chart for the comprehensive analysis of ICDR&CSRDEGs is shown in Fig. 1.

### Merging of GEO datasets for LIHC

**Fig. 2:**
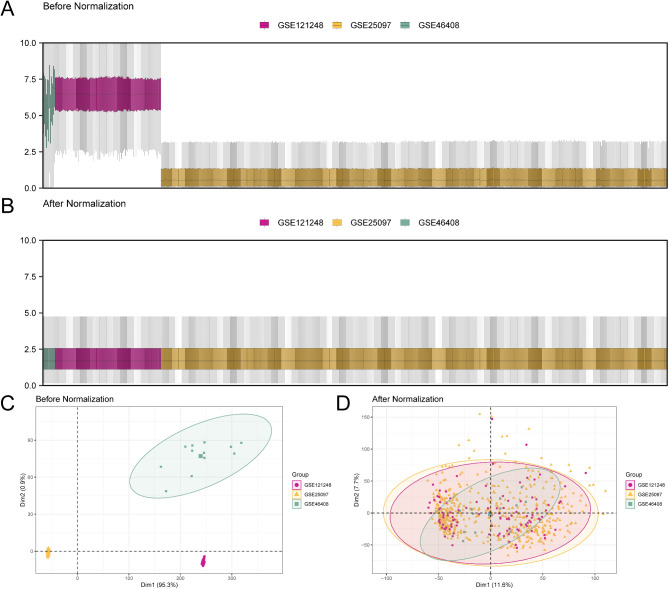
Batch effect removal of GSE25097, GSE46408, and GSE121248. (**A**) Box plot of dataset distribution before batch effect removal. (**B**) Box plot of the combined GEO dataset distribution after batch processing. (**C**) PCA plot of the datasets before debatching. (**D**) PCA map of the combined GEO datasets after batch processing. The liver hepatocellular carcinoma (LIHC) dataset GSE25097 is indicated in yellow, GSE46408 in green, and GSE121248 in red. PCA, Principal Component Analysis; GEO, Gene Expression Omnibus.

We used the R package sva to remove batch effects from the GSE25097, GSE46408, and GSE121248 datasets, which were then integrated for further analysis. The TCGA-LIHC dataset was analyzed separately without batch effect removal. Boxplots and principal component analysis (PCA) plots were used to assess the datasets before and after the removal of batch effects (Fig. 2A–D). The results of the grouping of boxplots and PCA plots showed that batch effects in the LIHC dataset samples were largely eliminated after this process.

### ICDR&CSRDEGs

**Fig. 3:**
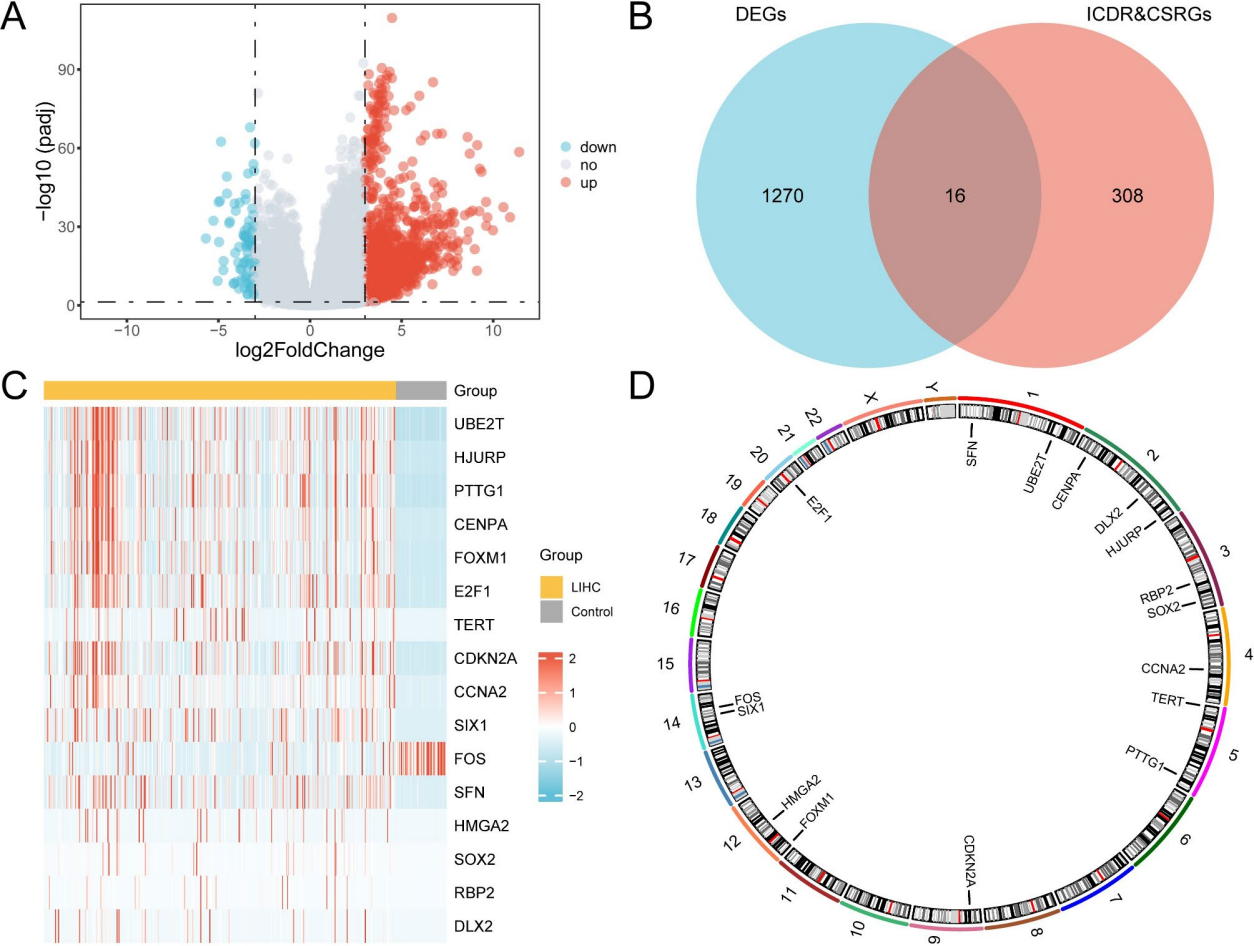
Differential gene expression analysis for TCGA-LIHC datasets. (**A**) Volcano plot of differential gene expression analysis between the LIHC and control groups in TCGA-LIHC dataset. (**B**) DEGs and Venn diagram of ICDR&CSRGs in TCGA-LIHC dataset. (**C**) Heatmap of ICDR&CSRDEGs in TCGA-LIHC dataset. (**D**) Chromosomal localization map of ICDR&CSRDEGs. TCGA, The Cancer Genome Atlas; LIHC, Liver Hepatocellular Carcinoma; DEGs, Differentially Expressed Genes; ICDR&CSRGs, Immunogenic Cell Death-Related and Cellular Senescence-Related Genes; ICDR&CSRDEGs, Immunogenic Cell Death-Related and Cellular Senescence-Related Differentially Expressed Genes. Control group (gray) and LIHC group (yellow).

TCGA-LIHC dataset was divided into LIHC and control groups. Differential gene expression between the groups was analyzed using the DESeq2 R package. The analysis identified 1,286 DEGs with |logFC| > 3 and adjusted p-value (p.adj) < 0.05, comprising 1,205 upregulated (logFC > 3) and 81 downregulated (logFC < -3) genes, as shown in the volcano plot in Fig. 3A.

ICDR&CSRDEGs were identified by finding the intersection of DEGs with |logFC| > 3 and p.adj < 0.05 and ICDR&CSRGs. A Venn diagram (Fig. 3B) illustrates the identification of the 16 ICDR&CSRDEGs, namely, *UBE2T*,* HJURP*,* PTTG1*,* CENPA*,* FOXM1*,* E2F1*,* TERT*,* CDKN2A*,* CCNA2*,* SIX1*,* FOS*,* SFN*,* HMGA2*,* SOX2*,* RBP2*, and *DLX2*.

A heatmap generated with the R package pheatmap (Fig. 3C) was used to visualize the expression differences of ICDR and CSRDEGs across different sample groups in TCGA-LIHC dataset. The chromosomal locations of the 16 ICDR&CSRDEGs were analyzed and visualized with the R package RCircos, producing a chromosomal localization map (Fig. 3D). The map revealed that numerous ICDR&CSRDEGs, such as *CENPA*, *DLX2*, and *HJURP*, are situated on chromosome 2.

### GO and KEGG pathway enrichment analyses of LIHC

GO and KEGG pathway enrichment analyses were performed to explore the relationships between the 16 ICDR&CSRDEGs and LIHC, focusing on biological processes, cellular components, molecular functions, and pathways. The results are detailed in Table 1.

**Table 1:** Results of GO and KEGG enrichment analyses for ICDR&CSRDEGs.

ONTOLOGY	ID	GeneRatio	BgRatio	*p*-value	*p*.adj	q-value
BP	GO:0044772	6/16	440/18,800	1.04255 e-06	0.000654461	0.000352088
BP	GO:0044843	5/16	255/18,800	1.70655 e-06	0.000654461	0.000352088
BP	GO:0045786	5/16	387/18,800	1.30495 e-05	0.002804538	0.001508789
BP	GO:1902806	4/16	188/18,800	1.60455 e-05	0.002804538	0.001508789
BP	GO:1901987	5/16	415/18,800	1.82825 e-05	0.002804538	0.001508789
CC	GO:0000792	3/16	69/19,594	2.26488 e-05	0.000405066	0.000119921
CC	GO:0032993	4/16	220/19,594	2.53166 e-05	0.000405066	0.000119921
CC	GO:0005667	4/16	483/19,594	0.000524321	0.005592763	0.001655752
CC	GO:0098687	3/16	366/19,594	0.003021249	0.024169991	0.007155589
CC	GO:0000776	2/16	146/19,594	0.006179102	0.029798215	0.00882184
MF	GO:0001216	5/16	466/18,410	3.52655 e-05	0.002539114	0.001113646
MF	GO:0001221	3/16	108/18,410	0.000104002	0.003744059	0.001642131
MF	GO:0030291	2/16	33/18,410	0.000368074	0.007175706	0.00314724
MF	GO:0001223	2/16	40/18,410	0.000541821	0.007175706	0.00314724
MF	GO:0001228	4/16	462/18,410	0.000560525	0.007175706	0.00314724
KEGG	hsa05166	6/13	222/8164	5.52991 e-07	3.58245 e-05	2.53054 e-05
KEGG	hsa04110	5/13	126/8164	9.42749 e-07	3.58245 e-05	2.53054 e-05
KEGG	hsa04218	4/13	156/8164	8.01905 e-05	0.002031494	0.001434989
KEGG	hsa01522	3/13	98/8164	0.00043963	0.008352979	0.005900304
KEGG	hsa05219	2/13	41/8164	0.001853391	0.022834407	0.016129567

GO, Gene Ontology; BP, Biological Process; CC, Cellular Component; MF, Molecular Function; KEGG, Kyoto Encyclopedia of Genes and Genomes; ICDR&CSRDEGs, Immunogenic Cell Death-Related and Cellular Senescence-Related Differentially Expressed Genes.

**Fig. 4:**
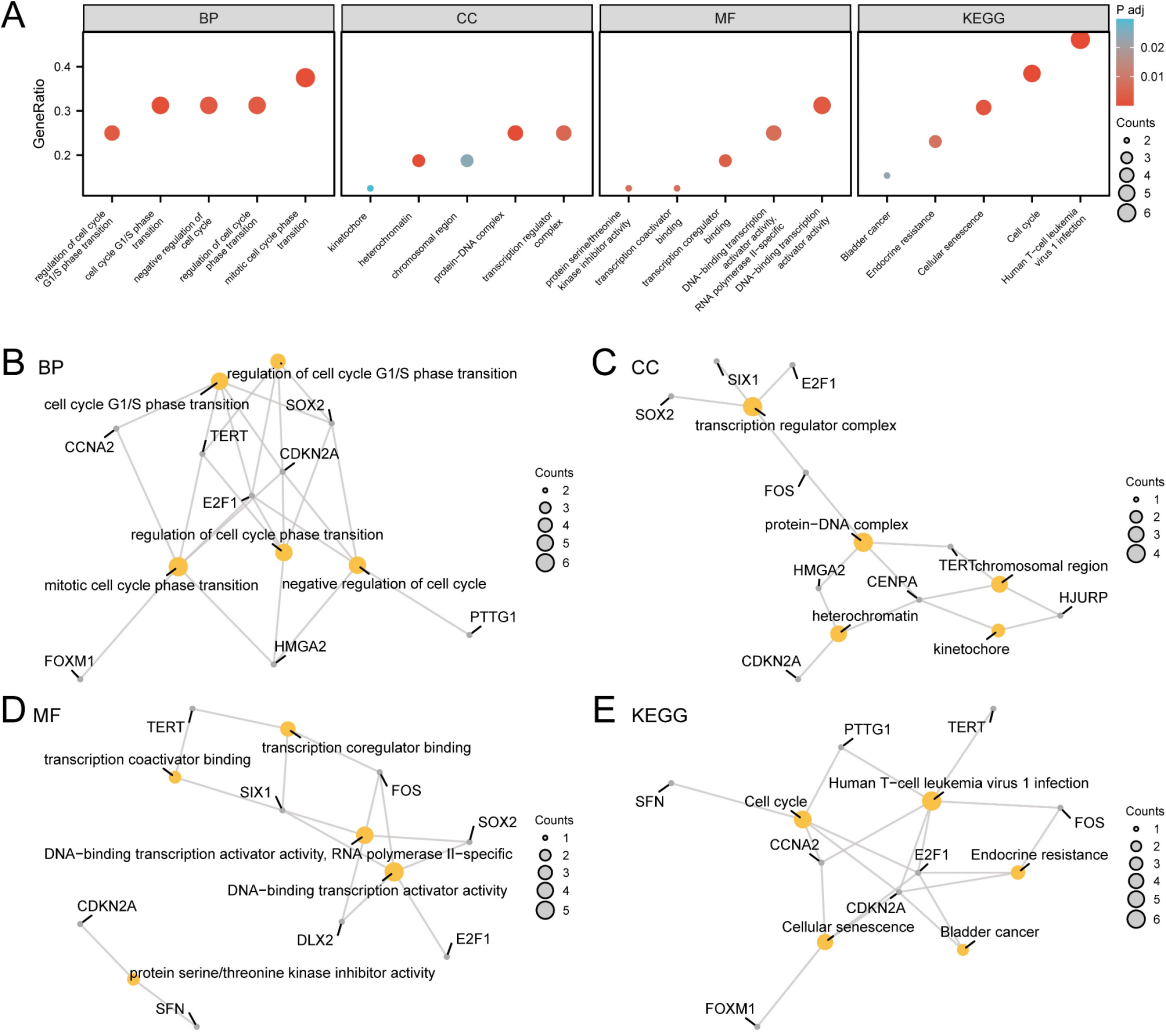
GO and KEGG enrichment analyses for ICDR&CSRDEGs. (**A**) Bubble plot of GO and KEGG pathway enrichment analyses of ICDR&CSRDEGs showing the BPs, CCs, MFs, and biological pathways (pathway); GO and KEGG terms on the abscissa. (**B**–**E**) The GO and KEGG pathway enrichment analysis results of ICDR&CSRDEGs. Network diagram showing BP (**B**), CC (**C**), MF (**D**), and KEGG pathways (**E**). Yellow nodes represent items, gray nodes represent molecules, and lines represent relationships between items and molecules. ICDR&CSRDEGs, Immunogenic Cell Death-Related and Cellular Senescence-Related Differentially Expressed Genes; GO, Gene Ontology; KEGG, Kyoto Encyclopedia of Genes and Genomes; BP, Biological Process; CC, Cellular Component; MF, Molecular Function. The screening criteria for GO and KEGG pathway enrichment analyses are p.adj < 0.05 and false discovery rate value (q-value) < 0.25; the Benjamini–Hochberg p-value correction method is applied.

The analysis revealed that the 16 ICDR&CSRDEGs were mainly enriched in cell cycle regulation and transition processes, such as the G1/S phase, negative regulation of the cell cycle, and mitotic phase transitions. These genes were linked to cellular components such as the kinetochore, heterochromatin, chromosomal region, protein–DNA complex, and transcription regulator complex. The enriched molecular functions comprised protein serine/threonine kinase inhibitor activity, transcription coactivator binding, DNA-binding transcription activator activity, and RNA polymerase II-specific DNA-binding transcription activator activity. These genes participated in biological pathways including bladder cancer, endocrine resistance, cellular senescence, cell cycle, and human T-cell leukemia virus 1 infection. Bubble plots were used to visualize the results of the GO and KEGG pathway enrichment analyses (Fig. 4A). Network diagrams illustrating biological processes, cellular components, molecular functions, and pathways were generated using GO and KEGG enrichment analyses (Fig. 4B–E).

### GSEA of LIHC

**Fig. 5:**
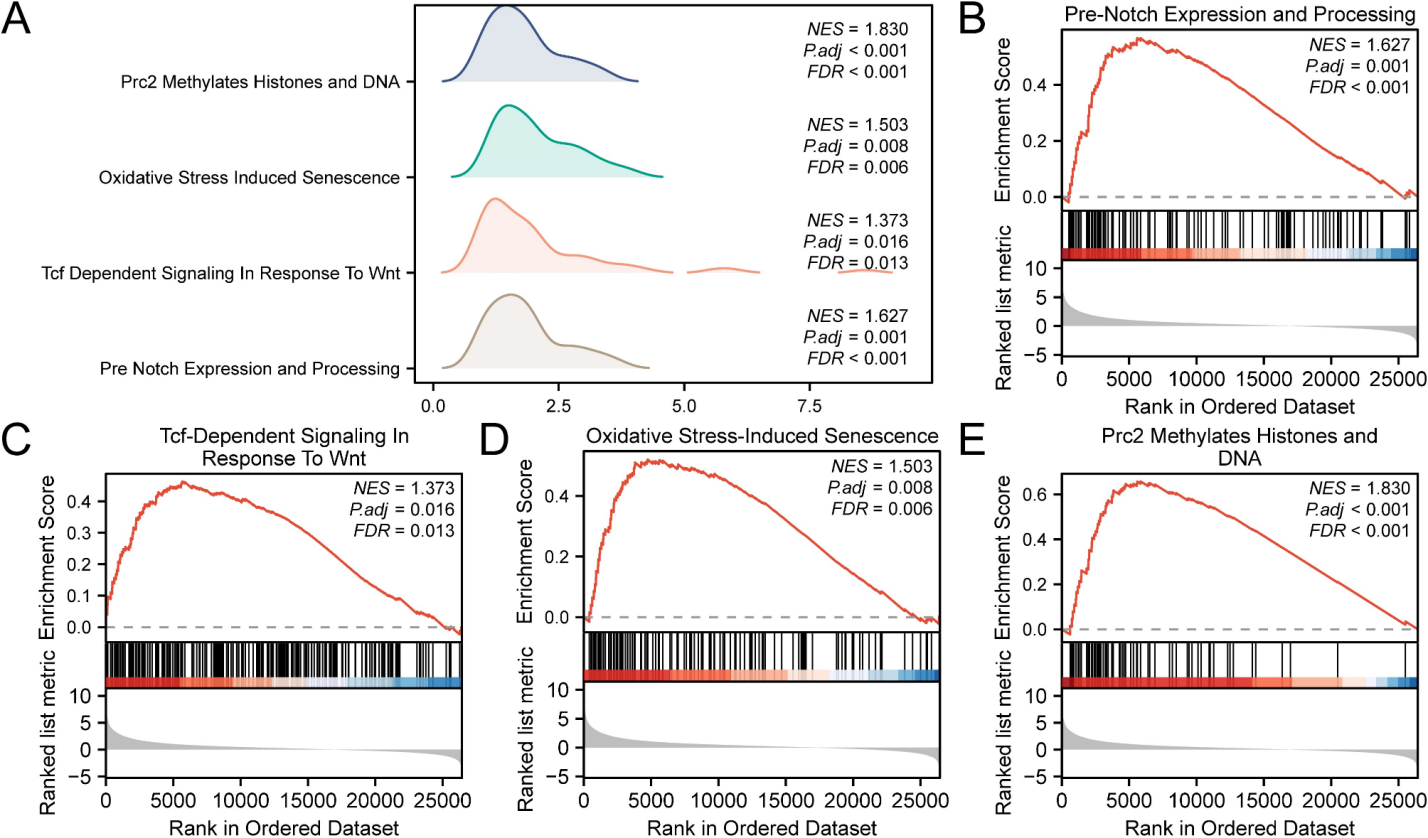
GSEA for TCGA-LIHC datasets. (**A**) GSEA of four biological functions or pathway bubble plots are presented for TCGA-LIHC dataset. (**B**–**E**) GSEA shows that LIHC is significantly enriched in pre-Notch expression and processing (**B**). Tcf-dependent signaling in response to Wnt (**C**), oxidative stress-induced senescence (**D**), and Prc2 methylation of histones and DNA (**E**). LIHC, Liver Hepatocellular Carcinoma; GSEA, Gene Set Enrichment Analysis. The screening criteria for GSEA are p.adj < 0.05 and false discovery rate value (q-value) < 0.25; the p-value correction method is Benjamini–Hochberg.

GSEA was performed to evaluate the influence of gene expression levels on LIHC progression using TCGA-LIHC dataset. We analyzed TCGA-LIHC dataset to explore the association between gene expression and their functions in biological processes, cellular components, and molecular functions (Fig. 5A), with detailed results in Table 2. The analysis demonstrated a notable enrichment of all genes in pertinent biological functions and signaling pathways, such as pre-Notch expression and processing. Figure 5B illustrates the Tcf-dependent signaling activated by Wnt (Fig. 5C), oxidative stress-induced senescence (Fig. 5D), and Prc2 methylation of histones and DNA (Fig. 5E).

**Table 2:** Results of GSEA for TCGA-LIHC datasets.

ID	SetSize	Enrichment score	NES	*p*-value	*p*.adj	q-value
REACTOME_PRE_NOTCH_EXPRESSION_AND_PROCESSING	103	0.56739	1.62748	6.48 e-05	1.16 e-03	8.93 e-04
REACTOME_TCF_DEPENDENT_SIGNALING_IN_RESPONSE_TO_WNT	226	0.46370	1.37310	2.02 e-03	1.64 e-02	1.27 e-02
REACTOME_OXIDATIVE_STRESS_INDUCED_SENESCENCE	117	0.52020	1.50315	7.76 e-04	7.89 e-03	6.10 e-03
REACTOME_PRC2_METHYLATES_HISTONES_AND_DNA	67	0.65783	1.82978	3.30 e-06	9.91 e-05	7.66 e-05
PID_PLK1_PATHWAY	46	0.76118	2.04124	6.77 e-09	4.39 e-07	3.39 e-07
REACTOME_RESOLUTION_OF_SISTER_CHROMATID_COHESION	125	0.66172	1.92092	1.00 e-10	1.23 e-08	9.51 e-09
PID_AURORA_B_PATHWAY	39	0.72948	1.91869	1.32 e-06	4.68 e-05	3.62 e-05
WP_GASTRIC_CANCER_NETWORK_1	27	0.76820	1.91080	7.92 e-06	2.05 e-04	1.59 e-04
REACTOME_MITOTIC_SPINDLE_CHECKPOINT	110	0.66266	1.90437	2.98 e-10	3.33 e-08	2.57 e-08
REACTOME_POLO_LIKE_KINASE_MEDIATED_EVENTS	16	0.83093	1.89761	2.37 e-05	4.89 e-04	3.78 e-04
REACTOME_UNWINDING_OF_DNA	12	0.87025	1.87720	2.22 e-05	4.63 e-04	3.58 e-04
REACTOME_CONDENSATION_OF_PROPHASE_CHROMOSOMES	68	0.67223	1.86828	2.53 e-07	1.11 e-05	8.61 e-06
REACTOME_ACTIVATION_OF_THE_PRE_REPLICATIVE_COMPLEX	33	0.72407	1.86612	1.85 e-05	3.98 e-04	3.08 e-04
REACTOME_ACTIVATION_OF_ATR_IN_RESPONSE_TO_REPLICATION_STRESS	37	0.71022	1.86265	2.54 e-05	5.12 e-04	3.96 e-04
REACTOME_ACTIVATED_PKN1_STIMULATES_TRANSCRIPTION_OF_AR_ANDROGEN_RECEPTOR_REGULATED_GENES_KLK2_AND_KLK3	61	0.67490	1.85803	9.55 e-07	3.51 e-05	2.71 e-05
REACTOME_DNA_METHYLATION	59	0.67426	1.85486	2.14 e-06	6.93 e-05	5.35 e-05
REACTOME_ASSEMBLY_OF_THE_ORC_COMPLEX_AT_THE_ORIGIN_OF_REPLICATION	63	0.66665	1.83933	1.33 e-06	4.68 e-05	3.62 e-05
REACTOME_GOLGI_CISTERNAE_PERICENTRIOLAR_STACK_REORGANIZATION	14	0.82451	1.82402	1.07 e-04	1.72 e-03	1.33 e-03
BIOCARTA_MCM_PATHWAY	18	0.78358	1.82082	1.30 e-04	1.96 e-03	1.51 e-03
REACTOME_MEIOTIC_RECOMBINATION	80	0.64659	1.82058	6.36 e-07	2.48 e-05	1.92 e-05

GSEA, Gene Set Enrichment Analysis; TCGA, The Cancer Genome Atlas; LIHC, Liver Hepatocellular Carcinoma; NES, Normalized Enrichment Score.

### GSVA of LIHC

**Fig. 6:**
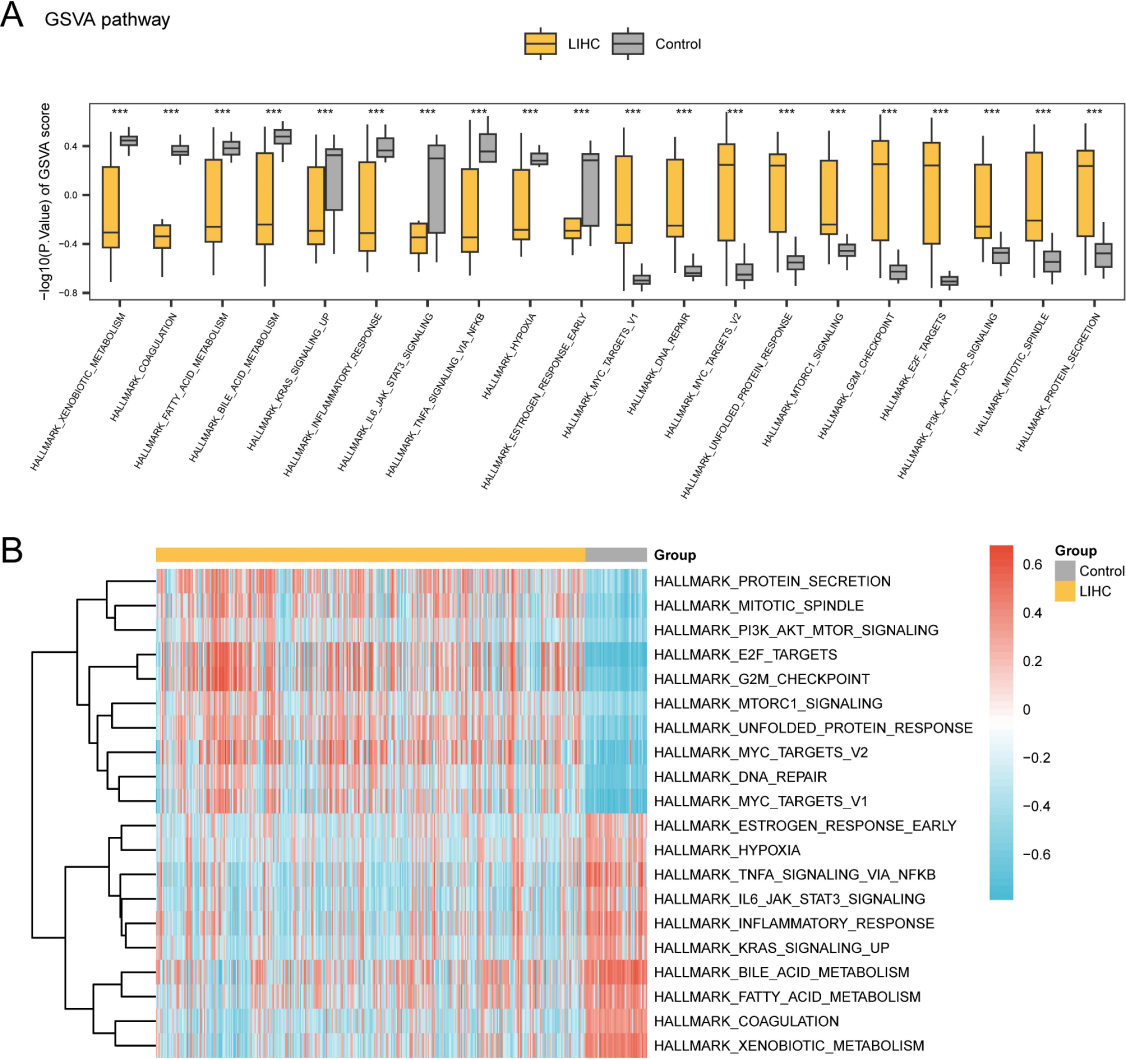
GSVA for TCGA-LIHC datasets. (**A**, **B**). Boxplot (**A**) and complex numerical heatmap (**B**) of group comparison of GSVA results in the LIHC and control groups. LIHC, Liver Hepatocellular Carcinoma; GSVA, Gene Set Variation Analysis. Gray is the Control group, and yellow is the LIHC group. ***p < 0.001. The screening criteria for GSVA are p.adj < 0.05 and positive or negative top 10 logFC.

GSVA was conducted to examine the differential expression of the h.all.v2023.1.Hs.symbols.gmt gene set between the LIHC and control groups within TCGA-LIHC dataset (Table 3). Pathways with p.adj < 0.05 and the top 10 highest and lowest logFC values were selected and illustrated using group comparison boxplots (Fig. 6A). GSVA revealed 20 pathways with significant differences (*p* < 0.001) between the LIHC and control groups, including xenobiotic metabolism, coagulation, bile acid metabolism, TNFα signaling via NF-κB, inflammatory response, fatty acid metabolism, KRAS signaling upregulation, IL-6 JAK-STAT3 signaling, hypoxia, early estrogen response, PI3K-AKT-mTOR signaling, mTORC1 signaling, protein secretion, mitotic spindle, DNA repair, unfolded protein response, MYC targets (versions 1 and 2), G2/M checkpoint, and E2F targets. A heatmap was used to analyze and visualize the differential expression of these pathways (Fig. 6B).

**Table 3:** Results of the GSVA for TCGA-LIHC datasets.

ID	logFC	AveExpr	*p*-value	*p*.adj
HALLMARK_XENOBIOTIC_METABOLISM	6.25 e-01	1.22 e-01	1.54 e-33	3.84 e-32
HALLMARK_COAGULATION	5.89 e-01	1.66 e-01	4.35 e-35	2.18 e-33
HALLMARK_BILE_ACID_METABOLISM	5.13 e-01	8.05 e-03	9.54 e-18	3.67 e-17
HALLMARK_TNFA_SIGNALING_VIA_NFKB	4.73 e-01	1.55 e-01	2.26 e-18	9.41 e-18
HALLMARK_INFLAMMATORY_RESPONSE	4.40 e-01	1.05 e-01	4.14 e-15	1.22 e-14
HALLMARK_FATTY_ACID_METABOLISM	4.04 e-01	3.83 e-02	4.28 e-14	1.13 e-13
HALLMARK_KRAS_SIGNALING_UP	3.57 e-01	1.30 e-01	7.97 e-14	1.99 e-13
HALLMARK_IL6_JAK_STAT3_SIGNALING	3.54 e-01	1.91 e-01	4.86 e-12	1.11 e-11
HALLMARK_HYPOXIA	3.39 e-01	1.22 e-01	1.01 e-14	2.81 e-14
HALLMARK_ESTROGEN_RESPONSE_EARLY	3.05 e-01	1.37 e-01	8.38 e-13	1.99 e-12
HALLMARK_PI3K_AKT_MTOR_SIGNALING	3.63 e-01	1.62 e-01	3.30 e-15	1.03 e-14
HALLMARK_MTORC1_SIGNALING	4.07 e-01	9.89 e-02	2.77 e-17	9.88 e-17
HALLMARK_PROTEIN_SECRETION	4.96 e-01	3.31 e-02	1.93 e-18	8.76 e-18
HALLMARK_MITOTIC_SPINDLE	5.17 e-01	8.94 e-02	7.76 e-20	3.88 e-19
HALLMARK_DNA_REPAIR	5.34 e-01	1.50 e-01	3.23 e-26	3.23 e-25
HALLMARK_UNFOLDED_PROTEIN_RESPONSE	5.75 e-01	2.93 e-02	2.14 e-27	2.68 e-26
HALLMARK_MYC_TARGETS_V1	5.89 e-01	1.51 e-01	3.96 e-24	2.83 e-23
HALLMARK_MYC_TARGETS_V2	6.40 e-01	4.51 e-02	9.72 e-24	6.08 e-23
HALLMARK_G2M_CHECKPOINT	6.72 e-01	3.04 e-02	3.10 e-25	2.59 e-24
HALLMARK_E2F_TARGETS	7.28 e-01	6.12 e-02	4.23 e-28	7.05 e-27

GSVA, Gene Set Variation Analysis; TCGA, The Cancer Genome Atlas; LIHC, Liver Hepatocellular Carcinoma.

### Somatic mutation (SM) and copy number variation (CNV) analysis

**Fig. 7:**
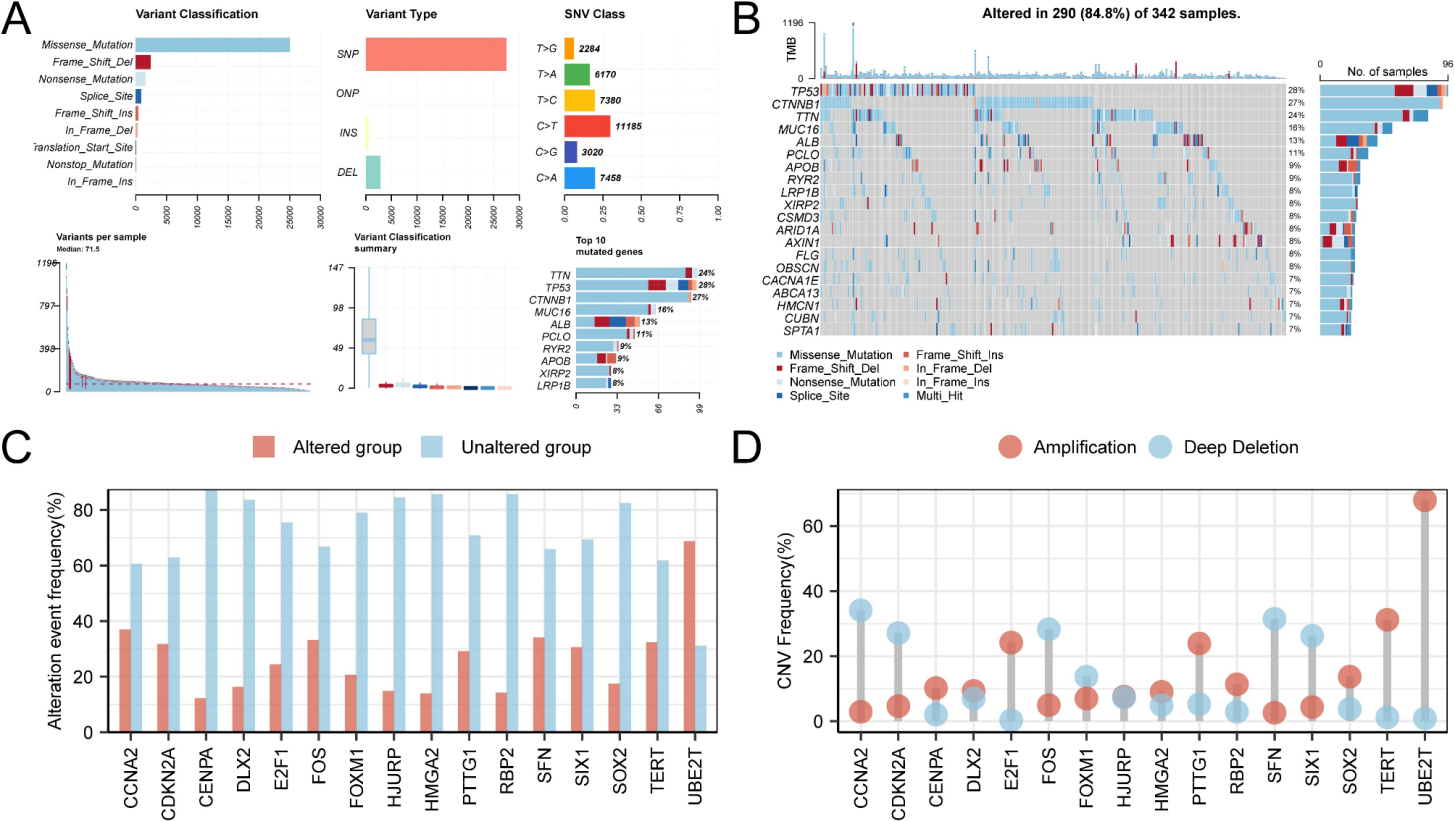
SM and CNV analysis. (**A**) SM presentation of all genes in LIHC samples from TCGA-LIHC dataset. (**B**) SM presentation (top 20 genes) of all genes in LIHC samples from TCGA-LIHC dataset. (**C**–**D**) ICDR&CSRDEGs with CNVs are shown in the LIHC samples from TCGA-LIHC dataset. TCGA, The Cancer Genome Atlas; LIHC, Liver Hepatocellular Carcinoma; ICDR&CSRDEGs, Immunogenic Cell Death-Related and Cellular Senescence-Related Differentially Expressed Genes; SM, Somatic Mutation; SNV, Single Nucleotide Variant; SNP, Single Nucleotide Polymorphism; CNV, Copy Number Variation.

We utilized the R package maftools to statistically analyze and visualize mutation data for all genes in TCGA-LIHC dataset (Fig. 7A). Nine primary types of SMs were identified, with missense mutations being the most prevalent. Single nucleotide polymorphisms (SNPs) were the dominant mutation types across all genes in the LIHC group, with C to T mutations being the most frequent single nucleotide variants. We examined the SM status of all genes in the LIHC samples, ranked them by mutation frequency, and visualized the top 20 most frequently mutated genes (Fig. 7B). *TP53* exhibited the highest mutation rate (28%).

We obtained and combined CNV data to examine the CNVs of 16 ICDR&CSRDEGs in LIHC samples from TCGA-LIHC dataset. The analysis, which was conducted using GISTIC2.0, revealed CNVs in 16 ICDR&CSRDEGs across 344 LIHC samples. The CNV status of the 16 genes—*UBE2T*, *HJURP*, *PTTG1*, *CENPA*, *FOXM1*, *E2F1*, *TERT*, *CDKN2A*, *CCNA2*, *SIX1*, *FOS*, *SFN*, *HMGA2*, *SOX2*, *RBP2*, and *DLX2*—was subsequently visualized (Fig. 7C–D).

### Creation of a prognostic risk model for LIHC

**Fig. 8:**
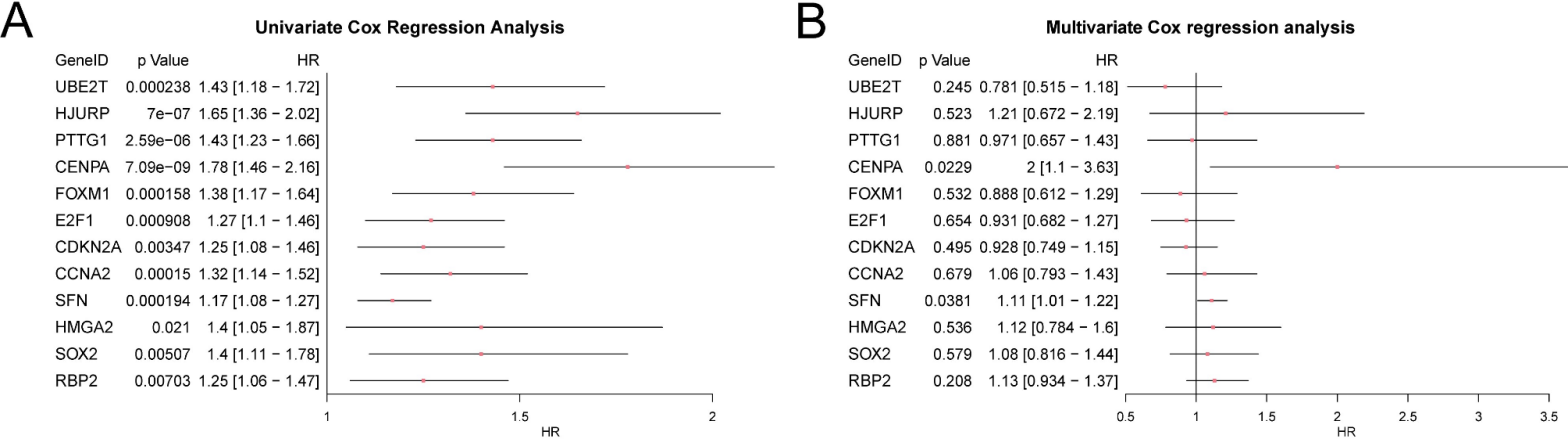
Univariable and multivariable Cox analyses. (**A**) Forest plot of 16 immunogenic cell death-related and cellular senescence-related differentially expressed genes (ICDR&CSRDEGs) in the univariate Cox regression model. (**B**) Forest plot of the multivariate regression model.

A prognostic risk model for LIHC was created using univariate Cox regression analysis on 16 ICDR&CSRDEGs. Variables with a p-value under 0.10 from this analysis were displayed in a forest plot (Fig. 8A). The univariate Cox regression model identified 12 ICDR&CSRDEGs as significant, namely, *UBE2T*,* HJURP*,* PTTG1*,* CENPA*,* FOXM1*,* E2F1*,* CDKN2A*,* CCNA2*,* SFN*,* HMGA2*,* SOX2*, and *RBP2*. A multivariate Cox regression analysis was performed on these genes to evaluate their prognostic significance in the LIHC risk model (Table 4), with the results displayed in a forest plot (Fig. 8B). A risk score was derived from the risk coefficients of the multivariate Cox regression analysis and calculated using the following formula:

$$\begin{gathered} {\text{risk}}Score~ = UBE2T*\left( { - 0.247} \right) + HJURP*\left( {0.192} \right) + PTTG1*\left( { - 0.030} \right) + CENPA*\left( {0.693} \right) \hfill \\ \quad \quad \quad \quad \quad \quad + FOXM1*\left( { - 0.119} \right) + E2F1*\left( { - 0.0713} \right) + CDKN2A*\left( { - 0.075} \right) + CCNA2*\left( {0.062} \right) \hfill \\ \quad \quad \quad \quad \quad \quad + SFN*\left( {0.103} \right) + HMGA2*\left( {0.112} \right) + SOX2*\left( {0.080} \right) + RBP2*\left( {0.123} \right) \hfill \\ \end{gathered}$$

**Table 4:** Results of the univariate and multivariate Cox regression analyses for TCGA-LIHC datasets.

Characteristics	Total (*N*)	Univariate analysis		Multivariate analysis	
		Hazard ratio (95% CI)	*p*-value	Hazard ratio (95% CI)	*p*-value
Sex	349	0.760 (0.523–1.10)	0.152		
Female	111			Reference	
Male	238			0.887 (0.602–1.306)	0.544
Age	349	1.25 (0.862–1.80)	0.241		
≤ 60 years	171			Reference	
> 60 years	178			1.301 (0.890–1.902)	0.174
Stage	349	1.42 (0.868–2.31)	**< 0.001**		
Stage I	173			Reference	
Stage II	86			1.164 (0.706–1.920)	0.551
Stage III	85			1.906 (1.217–2.985)	**0.005**
Stage IV	5			4.384 (1.321–14.551)	**0.016**
Risk score	349	2.72 (2.04–3.63)	**< 0.001**	2.427 (1.789–3.292)	**< 0.001**

TCGA, The Cancer Genome Atlas; LIHC, Liver Hepatocellular Carcinoma; CI, Confidence Interval.

Significant values are in [bold].

### Analysis of prognostic risk models for hepatocellular carcinoma

**Fig. 9:**
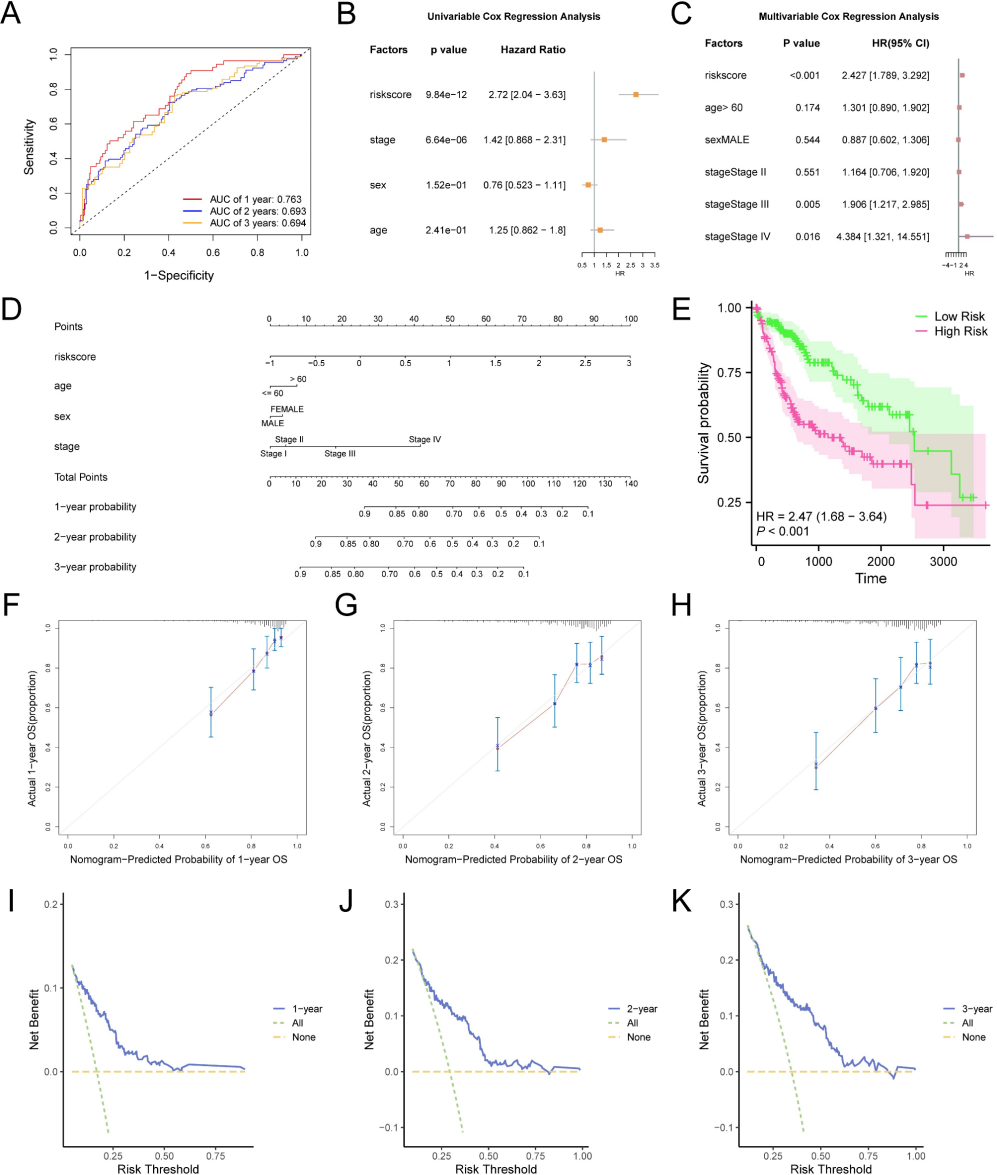
Prognostic analysis. (**A**) Time-dependent ROC curves at 1, 2, and 3 years. (**B**–**C**) Forest plot of the risk score and clinical information in the univariate Cox regression model (**B**) and multivariate Cox regression model (**C**). (**D**) Nomogram of the risk score and clinical information in the univariate and multivariate Cox regression model. (**E**) Kaplan–Meier curves are plotted according to the risk score. (**F**–**H**) 1- (**F**), 2- (**G**), and 3-year (**H**) calibration curves of the prognostic risk model for LIHC. (**I**–**K**) DCA curves at 1 (**I**), 2 (**J**), and 3 years (**K**) for the prognostic risk model for LIHC. LIHC, Liver Hepatocellular Carcinoma; ROC, Receiver Operating Characteristic Curve; AUC, Area Under the Curve; OS, Overall Survival; DCA, Decision Curve Analysis. P-value < 0.001, highly statistically significant. AUC between 0.5 and 0.7 indicates low accuracy; AUC between 0.7 and 0.9 indicates certain accuracy.

Figure 9A illustrates a time-dependent receiver operating characteristic (ROC) curve that was produced using the risk score for LIHC in TCGA-LIHC dataset. The LIHC prognostic risk model demonstrated moderate accuracy at 1 year (AUC between 0.7 and 0.9) and reduced accuracy at 2 and 3 years (AUC between 0.5 and 0.7).

Based on the median risk score from the prognostic model, LIHC samples were categorized into high- and low-risk groups. A univariate Cox regression analysis was conducted on the risk score and clinical data from LIHC samples, incorporating variables with a p-value < 0.10 into a subsequent multivariate Cox regression analysis. Forest plots (Fig. 9B–C) presented the results of univariate and multivariate analyses. The findings indicated that the risk score, in conjunction with clinical variables such as age, sex, and pathological stage, were significant (*p* < 0.05).

A nomogram was developed through univariate and multivariate Cox regression analyses to evaluate the prognostic risk model for LIHC, highlighting the association between the risk score and clinical variables in the samples (Fig. 9D). The findings indicated that the utility of the risk score in the LIHC prognostic risk model surpassed that of the other variables, whereas the utility of sex was low.

A Kaplan–Meier curve analysis evaluated overall survival in LIHC samples using median risk score grouping (Fig. 9E). The findings revealed a significant difference in overall survival between high- and low-risk groups (*p* < 0.001).

Calibration analyses for 1-, 2-, and 3-year outcomes were conducted and visualized using calibration curves (Fig. 9F–H). In these curves, the horizontal axis represents the model’s predicted survival probability, whereas the vertical axis shows the observed survival probability. The predictive accuracy is enhanced when the model-predicted lines for different time points closely align with the ideal gray line. The prognostic risk model for LIHC showed strong clinical predictive accuracy at 1, 2, and 3 years.

Decision curve analysis (DCA) was conducted over 1, 2, and 3 years to assess the clinical utility of the LIHC prognostic risk model, as illustrated in the DCA curves (Fig. 9I–K). The model’s effectiveness is indicated by the x-value range where its line consistently exceeds the “All positive” and “All negative” lines. An increased range of x values corresponds with improved model performance. The findings revealed that the LIHC prognostic risk model offered superior clinical predictive accuracy.

### Immune-cell infiltration analysis

**Fig. 10:**
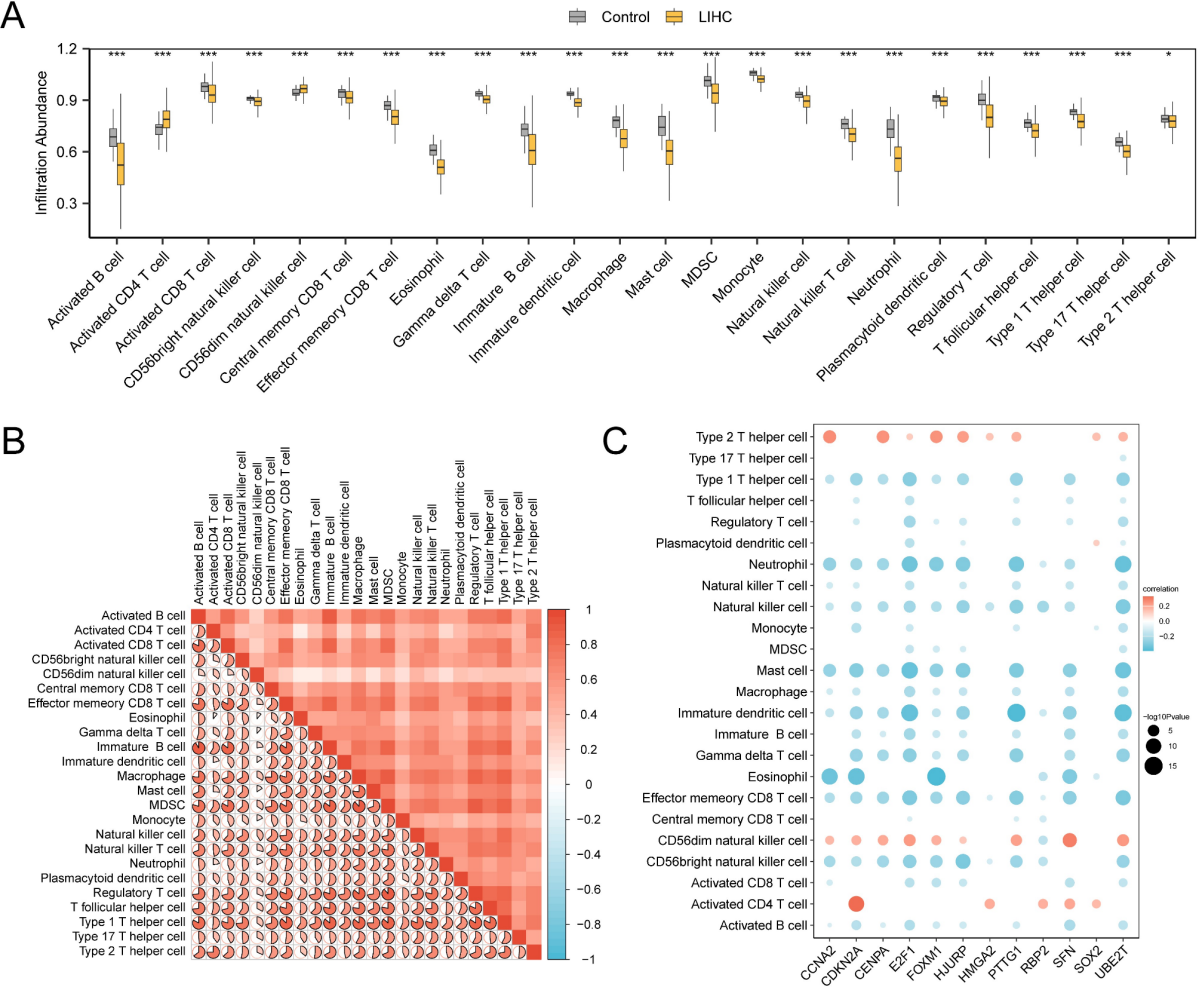
TCGA-LIHC immune-cell infiltration analysis using the ssGSEA algorithm. (**A**) Plot of grouping comparison of immune cells in the LIHC and control groups. (**B**) Correlation heatmap of immune-cell infiltration abundance with significant difference in the group comparison map in TCGA-LIHC dataset. (**C**) Heatmap of correlation between model genes and infiltration abundance of 24 immune cells in TCGA-LIHC dataset. ssGSEA, Single-Sample Gene Set Enrichment Analysis; LIHC, Liver Hepatocellular Carcinoma; TCGA, The Cancer Genome Atlas. *p < 0.05; ***p < 0.001. The absolute value of correlation coefficient (r value) below 0.3 indicates weak or no correlation, between 0.3 and 0.5 indicated weak correlation, between 0.5 and 0.8 indicated moderate correlation, and more than 0.8 indicated strong correlation. Yellow indicates the LIHC group and gray the control group.

We utilized the ssGSEA algorithm to evaluate the abundance and correlation of immune-cell infiltration across 28 immune-cell types using samples from TCGA-LIHC dataset. Using immune-cell infiltration analysis data from TCGA-LIHC dataset, we generated plots to compare immune-cell infiltration between the LIHC and control groups. Figure 10A reveals significant differences (*p* < 0.05) in the abundance of 24 immune cells between the LIHC and control groups. The infiltration abundance of various immune cells, including B cells, T cells (CD4^+^, CD8^+^, gamma-delta, regulatory, and follicular helper), natural killer cells (CD56bright, CD56dim), memory CD8^+^ T cells (central, effector), eosinophils, immature B and dendritic cells, macrophages, mast cells, myeloid-derived suppressor cells, monocytes, neutrophils, plasmacytoid dendritic cells, and type 1 and 17 T-helper cells, showed significant differences between the LIHC and control groups (*p* < 0.001). The groups showed a significant difference in type 2 T helper-cell infiltration abundance (*p* < 0.05).

A correlation heatmap was generated to depict the relationships among the infiltration abundance of 24 distinct immune cells in TCGA-LIHC dataset samples (Fig. 10B). This analysis revealed a positive correlation across all immune cell types, with particularly strong positive correlations observed between activated and immature B cells and between MDSCs and Tregs.

Figure 10C illustrates a correlation heatmap showing the relationship between 12 genes linked to the prognostic risk model and the abundance of 24 immune cell types in TCGA-LIHC dataset. *CDKN2A* exhibited the strongest positive correlation with activated CD4 T-cell infiltration, whereas *FOXM1* had the most pronounced negative correlation with eosinophil infiltration.

### Analysis of differential gene expression in the prognostic risk model

**Fig. 11:**
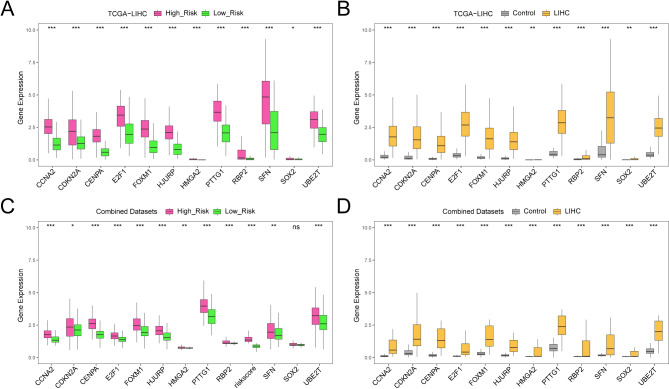
Expression difference analysis for model genes. (**A**) Boxplot of prognostic risk model-related genes (model genes) in LIHC high- and low-risk groups in TCGA-LIHC dataset. (**B**) Boxplots of prognostic risk model-related genes in the LIHC and control groups in TCGA-LIHC dataset. (**C**) Boxplots of prognostic risk model-associated genes in LIHC samples from combined datasets in the high- and low-risk groups. (**D**) Boxplots of prognostic risk model-associated genes in the LIHC and control groups in combined datasets. TCGA, The Cancer Genome Atlas; LIHC, Liver Hepatocellular Carcinoma. High-risk group is indicated in red and low-risk group in green. Orange-yellow indicates the LIHC group and gray the control group. *p < 0.05; **p < 0.01; ***p < 0.001.

Boxplots depicted the expression differences of 12 prognostic risk model-related genes between high- and low-risk groups in TCGA-LIHC dataset and LIHC samples. Our analysis identified notable variations in the expression levels of all 12 model genes (*p* < 0.05, Fig. 11A). Eleven genes (*UBE2T*,* HJURP*,* PTTG1*,* CENPA*,* FOXM1*,* E2F1*,* CDKN2A*,* CCNA2*,* SFN*,* HMGA2*, and *RBP2*) showed strong differences in expression levels (*p* < 0.001). *SOX2* showed a significant difference in expression level as well (*p* < 0.05).

We analyzed the differential expression of model genes between LIHC and control samples using TCGA-LIHC dataset. Boxplots illustrated significant expression differences for all 12 model genes between LIHC and control groups (*p* < 0.05) (Fig. 11B). Twelve genes demonstrated significant differences, with *UBE2T*,* HJURP*,* PTTG1*,* CENPA*,* FOXM1*,* E2F1*,* CDKN2A*,* CCNA2*,* SFN*, and *RBP2* showing p-values below 0.001 and *SOX2* and *HMGA2* showing p-values below 0.01.

Differences in the expression of model genes related to the prognostic risk models were examined in LIHC samples from the combined datasets. Gene expression data and multivariate Cox analysis coefficients from the LIHC prognostic model were used to calculate risk scores, classifying samples into high- and low-risk groups based on the median. Figure 11C presents a boxplot illustrating the differential expression analysis between the groups, highlighting significant differences (*p* < 0.05) in 11 model genes: *UBE2T*,* HJURP*,* PTTG1*,* CENPA*,* FOXM1*,* E2F1*,* CCNA2*, and *RBP2* exhibited the strongest differences in expression levels (*p* < 0.001), whereas *HMGA2* and *SFN* showed strong significant differences (*p* < 0.01) and *CDKN2A* showed significant differences (*p* < 0.05).

Boxplots illustrated the differential expression of the 12 model genes between LIHC and control samples in the combined datasets (Fig. 11D). Significant differences (*p* < 0.001) were observed in the expression of all 12 model genes—*UBE2T*,* HJURP*,* PTTG1*,* CENPA*,* FOXM1*,* E2F1*,* CDKN2A*,* CCNA2*,* SFN*,* HMGA2*,* SOX2*, and *RBP*—between the LIHC and control groups.

## Discussion

In this study, we investigated the differential expression of genes associated with ICD and cellular senescence in LIHC to clarify their impact on disease progression and prognosis. LIHC is notably aggressive with a poor prognosis, significantly impacting patient quality of life and survival rates^1,10^. The high mortality rate associated with LIHC underscores the urgent need for novel therapeutic strategies and improved diagnostic tools. With the development of precision medicine, the value of molecular targets in the treatment, diagnosis, and prediction of cancer has received increasing attention. Although many biomarker-based LIHC prediction models have been reported, most of them are single-omic models, and their predictive ability is not satisfactory.

The role of ICD and cellular senescence in tumor progression is not fully understood, although some studies suggest that they exert antitumor effects^11^. ICD induces immune responses targeting tumor cells through cell death, whereas cellular senescence involves permanent cell cycle arrest, affecting both tumor suppression and progression^3,4^. Through the integration of data from various GEO datasets and TCGA-LIHC dataset, we identified 16 key ICDR&CSRDEGs that were differentially expressed in LIHC: *UBE2T*,* HJURP*,* PTTG1*,* CENPA*,* FOXM1*,* E2F1*,* TERT*,* CDKN2A*,* CCNA2*,* SIX1*,* FOS*,* SFN*,* HMGA2*,* SOX2*,* RBP2*, and *DLX2*. Our study highlights the pivotal role of ICDR&CSRDEGs in the tumor microenvironment, suggesting their potential as prognostic biomarkers and therapeutic targets. The development and validation of a prognostic risk model using these genes highlight their clinical significance and provide new perspectives on personalized treatment strategies for patients with LIHC.

Enrichment analysis of the 16 ICDR&CSRDEGs indicated their significant roles in various biological processes and pathways. The GO analysis highlighted the regulation of the G1/S phase transition in the cell cycle, a critical factor in cell proliferation and tumor growth, aligning with the findings of previous studies that identified cell cycle dysregulation as a hallmark of cancer, including LIHC^12–15^. This regulation is vital to control cell proliferation and tumor growth and a potential target for cancer therapy^13,16^. The KEGG pathway analysis identified significant enrichment in pathways in cancers such as bladder cancer, suggesting potential shared mechanisms among different cancer types. The GSEA highlighted the significant enrichment of genes in the pre-Notch expression and processing pathway, crucial for cell differentiation, proliferation, and apoptosis. The Notch signaling pathway influences cancer development, including LIHC, by affecting the tumor microenvironment and immune response^17^. Additionally, three other pathways are closely associated with tumor occurrence and progression. The Wnt-activated Tcf-dependent signaling pathway is essential for cancer stem cell renewal, proliferation, and differentiation, playing a significant role in tumorigenesis and therapeutic responses^18^.

GSVA revealed 20 pathways with significant differences between the LIHC and control groups, highlighting the IL6-JAK-STAT3 and PI3K-AKT-MTOR signaling pathways, which are closely associated with LIHC development. We also investigated mutations in the 16 ICDR&CSRDEGs and analyzed their correlation with tumor mutation burden. *TP53* showed a mutation rate of 28% in LIHC samples, which aligns with its recognized role as a frequently mutated tumor suppressor in various cancers. *TP53* mutations contribute to poor prognosis and therapy resistance, underscoring their role in LIHC pathogenesis^19^.

Through further data analysis, we obtained 12 model genes from the 16 ICDR&CSRGDEGs: *UBE2T*,* HJURP*,* PTTG1*,* CENPA*,* FOXM1*,* E2F1*,* CDKN2A*,* CCNA2*,* SFN*,* HMGA2*,* SOX2*, and *RBP2.* Next, we constructed the prognostic model based on 12 model genes and the risk coefficient of multivariate Cox regression analysis. The prognostic risk model for LIHC demonstrated a good clinical prediction effect at 1-, 2-, and 3-year intervals. A prognostic risk model incorporating 12 markers, including *UBE2T*,* HJURP*,* PTTG1*, and *CENPA*, highlights the importance of these genes in predicting patient outcomes. The time-dependent ROC curves highlight the risk model’s potential effectiveness in clinical applications, particularly for short-term prognosis.

Model genes were significantly upregulated in LIHC, playing key roles in tumor progression, prognosis, and potential therapeutic interventions, which is consistent with previous study findings. *UBE2T*, which is involved in ubiquitination and DNA damage repair, contributes to genomic stability, and its overexpression has been linked to poor prognosis in various cancers, including LIHC, suggesting a role in tumor progression and therapy resistance. *HJURP*, which is essential for maintaining centromeric chromatin, enhances tumor cell proliferation and is correlated with aggressive tumor characteristics and unfavorable clinical outcomes in LIHC. *PTTG1*, which encodes a securin protein involved in mitosis regulation, is often overexpressed in LIHC and linked to tumor aggressiveness and apoptosis resistance. *CENPA*, which is crucial for centromeric function and genome stability, is significantly upregulated in LIHC, leading to chromosomal instability and tumorigenesis. *FOXM1*, encoding a transcription factor that regulates cell cycle and DNA repair, is markedly upregulated in LIHC and is linked to poor clinical outcomes and increased tumor aggressiveness. *E2F1*, encoding a transcription factor, contributes to tumor growth and metastasis in multiple human cancers, including hepatocellular carcinoma^20^. Luo et al.^21^reported that *CDKN2A* was overexpressed in liver cancer, which correlates with poor prognosis and decreased immune-cell infiltration in hepatocellular carcinoma. *CCNA2*,* SFN*,* HMGA2*,* SOX2*, and *RBP2* have been identified as significant biomarkers for cancer diagnosis and prognosis, particularly in liver cancer^22–26^. High expression levels of these genes are correlated with poor staging, higher tumor grades, and unfavorable survival outcomes. Their inclusion in prognostic models highlights their potential as predictive biomarkers and therapeutic targets, emphasizing their roles in tumor immunity and progression.

In this study, *CENPA* and *SFN* were identified as independent prognosis-related genes, suggesting that they may play important roles in the occurrence and progression of LIHC. Previous studies have shown that CENPA, as a centriole-associated protein, may affect LIHC development by regulating chromosome stability and promoting mitotic abnormalities^27,28^. CENPA overexpression may cause chromosome missegregation, promote genomic instability, and ultimately promote tumor progression. In addition, CENPA may also affect cell division and tumor growth by interacting with other key cell cycle proteins.

SFN, as a cell cycle regulator, has an important regulatory role in a variety of cancers^29^. Studies have shown that SFN can improve the tolerance of tumor cells to external stress (such as chemotherapy and oxidative stress) by regulating DNA damage repair mechanisms. In addition, SFN may promote the proliferation, migration, and invasion of LIHC cells by activating the PI3K/AKT or MAPK signaling pathways. These effects make SFN a potential therapeutic target in LIHC, and its abnormal expression may be closely related to the poor patient prognosis.

Based on the key roles of CENPA and SFN in LIHC progression, future studies can further explore the molecular regulatory network of these two genes in LIHC pathogenesis, study their functions in the tumor microenvironment, and evaluate their application value in personalized treatment. In addition, verifying their protein-level expressional changes and exploring their relationships with immune infiltration, drug resistance, and other tumor-related factors will facilitate our understanding of their roles in LIHC development.

Emerging evidence indicates a strong association between immune cells, the microenvironment, and tumor development. The immune-cell infiltration analysis in our study provides crucial insights into the tumor microenvironment of LIHC. We utilized ssGSEA to evaluate immune-cell abundance across samples, identifying notable differences in immune-cell infiltration between high- and low-risk groups. Notable differences were identified in the infiltration of 24 immune cell types between the LIHC and control groups (*p* < 0.05). Positive correlations existed among all immune cells, with strong associations between activated and immature B cells and between MDSCs and Tregs, which was associated with impaired antitumor immunity. Among the 12 model genes, *CDKN2A* was positively correlated with activated CD4^+^ T-cell infiltration, whereas *FOXM1* was negatively correlated with eosinophil infiltration. CD8^+^ T cells, macrophages, and Tregs exhibited distinct infiltration patterns that are crucial for tumor immunity and progression. CD8^+^ T cells are critical for antitumor immunity, because they eliminate cancer cells directly through the release of cytotoxic granules and cytokines^30^. Tregs are known for their immunosuppressive functions that can inhibit effective antitumor immune responses^31–33^. The elevated presence of Tregs in the high-risk group suggests a more immunosuppressive tumor microenvironment, which may promote tumor growth and resistance to immunotherapy. These findings align with previous study findings highlighting the prognostic significance of immune-cell infiltration in various cancers, including LIHC^34–36^.

The integration of our immune-cell infiltration analysis with the identified ICDR&CSRDEGs offers a thorough insight into the immune landscape in LIHC. This knowledge is crucial to develop targeted immunotherapies and improve patient stratification based on immune profiles. Future research should aim to confirm these results in larger groups and investigate the therapeutic potential of altering immune-cell infiltration to boost antitumor immunity in patients with LIHC.

This study provides a new strategy for integrating ICDRGs and CSRGs to assess LIHC prognosis. Compared with existing studies, it introduces several innovations. First, we aggregated the TCGA-LIHC dataset and multiple GEO array datasets (GSE25097, GSE46408, and GSE121248) and performed strict data standardization and batch effect correction to obtain more reliable gene expression features.

We also constructed a comprehensive prognostic risk model based on ICDRGs and CSRGs and verified its predictive efficacy in the short- (1 year) and long-term (2–3 years). This approach differs from those of past single-model studies, which were limited to ICDRGs or CSRGs. This study also analyzed the immune cell infiltration characteristics of high- and low-risk groups to provide deeper insights into the LIHC immune microenvironment. We also combined SNV and CNV analyses to reveal the gene mutation characteristics that may affect LIHC prognosis.

This study systematically analyzed the functional characteristics of key genes based on the integration of data from multi-source public databases, combined with bioinformatics methods such as DESeq2 differential expression analysis, GO/KEGG functional enrichment, and GSEA/GSVA pathway enrichment analysis. It demonstrated good predictive performance and result stability in an independent validation set. However, the following limitations should be noted: Firstly, the current analysis is mainly limited to the transcriptome level, and the protein expression levels of key genes and their clinical relevance have not been verified through experimental methods such as Western blotting and immunofluorescence double-staining, which may affect the completeness of the molecular mechanism explanation. Secondly, although a preliminary validated prognostic model has been established, its clinical applicability and stability still need to be further verified in larger-scale multi-center external cohorts. These limitations mainly stem from the objective constraints of research resources and time costs at the present stage. Subsequent studies will focus on conducting functional verification experiments at the protein level (such as Western blotting and immunofluorescence double-staining) and multi-center prospective cohort studies to comprehensively enhance the biological plausibility and implementing clinical translational value of the research conclusions.

In conclusion, we effectively combined GEO datasets and TCGA data to identify DEGs associated with ICD and cellular senescence in LIHC. We developed a prognostic risk model with high predictive accuracy, validated through multiple statistical methods. Functional enrichment analyses identified biological processes and pathways linked to these genes. Analysis of immune-cell infiltration demonstrated a correlation between immune cell abundance and genes within the prognostic model. These findings enhance our comprehension of the molecular mechanisms in LIHC and indicate possible prognostic biomarkers and therapeutic targets. Future research should prioritize clinical validation and experimental studies to assess the applicability and biological significance of these findings.

## Methods

### Data download

We obtained the LIHC datasets GSE25097^37^, GSE46408^38^, and GSE121248^39^ from the GEO database^40^ using the GEOquery R package (Version 2.70.0, https://bioconductor.org/packages/release/bioc/html/GEOquery.html)^41^. Detailed information is provided in Table 5. All samples in these datasets originated from the liver tissues of Homo sapiens. Specifically, the GSE25097 dataset utilized the GPL10687 chip platform and comprised 268 LIHC and 243 control samples. The dataset GSE46408 utilized the GPL4133 chip platform, including six LIHC and six control samples. The chip platform for the GSE121248 dataset was GPL570, including 70 LIHC and 37 control samples. This study included all LIHC and control samples. The R package sva (Version 3.50.0, https://bioconductor.org/packages/release/bioc/html/sva.html)^42^ was employed for batch correction on datasets GSE25097, GSE46408, and GSE121248, producing the combined GEO datasets, including 344 LIHC and 286 control samples. The R package limma (Version 3.58.1, https://bioconductor.org/packages/limma/)^43^ was then used for normalization, probe annotation, and further standardization of the combined GEO Datasets.

**Table 5:** GEO microarray chip information.

	GSE25097	GSE46408	GSE121248
Platform	GPL10687	GPL4133	GPL570
Type	Array	Array	Array
Species	*Homo sapiens*	*Homo sapiens*	*Homo sapiens*
Tissue	Liver	Liver	Liver
Samples in the tumor group	268	6	70
Samples in the control group	243	6	37
Reference	PMID: 21955977	PMID: 23922981	PMID: 17975138

GEO, Gene Expression Omnibus.

TCGA-LIHC dataset was acquired from TCGA via TCGAbiolinks (Version 2.30.0, https://github.com/BioinformaticsFMRP/TCGAbiolinks)^44^ R package for further analysis. We analyzed 399 LIHC samples with comprehensive clinical data, comprising 349 tumor tissues (cancer group, LIHC) and 50 partially matched adjacent tissues (normal group, control). Data were normalized to the FPKM format with clinical information obtained from the UCSC Xena database^45^, as detailed in Table 6.

**Table 6:** Baseline table with LIHC patient characteristics.

Characteristics	Overall
Pathologic stage, n (%)	
Stage I	171 (49.3%)
Stage II	86 (24.8%)
Stages III and IV	90 (25.9%)
Sex, n (%)	
Female	121 (32.6%)
Male	250 (67.4%)
Age, n (%)	
≤ 60 years	177 (47.8%)
> 60 years	193 (52.2%)

LIHC, Liver Hepatocellular Carcinoma.

ICDRGs were sourced from the GeneCards database^46^ and existing literature. The GeneCards database provides extensive data on human genes. A search for “Immunogenic cell death” focusing on “Protein Coding” genes with a relevance score greater than 0 identified 63 ICDRGs. Similarly, 20 ICDRGs were obtained from the published literature^47^, using “Immunogenic cell death” as the search keywords in PubMed. After merging and removing duplicates, 79 ICDRGs were identified, as detailed in Table S1.

CSRGs were collected from the GeneCards database and published literature. “Cellular senescence” was used as a search keyword in GeneCards. Only “Protein Coding” and CSRGs with relevance scores > 5 were retained, and 63 CSRGs were obtained. Additionally, a PubMed search with the same keyword yielded 279 CSRGs from the literature^48^. After removing duplicates, 310 CSRGs were compiled. The detailed information is shown in Table S2. A total of 324 ICDR&CSRGs were obtained by merging and deduplication of ICDRGs and CSRGs (Table S3).

### ICDR&CSRDEGs

Using the DESeq2 R package (Version 1.42.0, https://github.com/thelovelab/DESeq2)^49^, differential expression analysis was conducted on samples from TCGA-LIHC dataset, categorized into LIHC and control groups. DEGs were identified with the criteria of |logFC| > 3 and p.adj < 0.05.Genes were classified as upregulated if logFC > 3 and p.adj < 0.05 and as downregulated if logFC < -3 and p.adj < 0.05.

To obtain ICDR&CSRDEGs associated with LIHC-expressed genes, TCGA-LIHC dataset was analyzed. Variance analysis was conducted on DEGs with absolute logFC > 3 and p.adj < 0.05. A Venn diagram was used to determine the overlap between these DEGs and ICDR&CSRGs, identifying the ICDR&CSRDEGs. Variance analysis results were visualized in a volcano plot using ggplot2 (Version 3.4.4, https://github.com/tidyverse/ggplot2) and heatmap using pheatmap; RCircos (Version 1.2.2, https://github.com/hzhanghenry/Rcircos) was used for chromosomal locations in R^50^.

### GO and KEGG pathway enrichment analyses of LIHC

GO analysis^51^ is widely utilized for large-scale functional enrichment studies, encompassing biological processes, cellular components, and molecular functions. The KEGG^52^ database offers comprehensive data on genomes, biological pathways, diseases, and drugs. The R package clusterProfiler (Version 4.10.0, https://bioconductor.org/packages/clusterProfiler/)^53^ conducted GO and KEGG pathway enrichment analyses on ICDR&CSRDEGs, deeming results statistically significant with a p.adj < 0.05 and q-value < 0.25, applying the Benjamini–Hochberg method for p-value correction.

### GSEA of LIHC

GSEA^54^ shows gene distribution trends in predefined sets within a phenotype-correlated ranked gene expression table to assess their phenotypic contribution. Genes from TCGA-LIHC dataset were categorized based on their median log fold change values. GSEA was performed on the dataset using the clusterProfiler R package, configured with a random seed of 2022, and gene set size constraints of 10 to 500. Gene sets were sourced from the Molecular Signatures Database (MSigDB) using the c2.cp.all.v2022.1.Hs.symbols.gmt collection, which includes 3,050 canonical pathways. GSEA significance was evaluated using the Benjamini–Hochberg correction for p-values, with thresholds of p.adj < 0.05 and a false discovery rate (q-value) < 0.25.

### GSVA of LIHC

GSVA^55^ is a nonparametric, unsupervised approach for evaluating gene set enrichment in microarray and transcriptomic datasets. It transforms a gene expression matrix from various samples into a gene set expression matrix, facilitating pathway enrichment analysis across samples. The GSVA R package (Version 1.50.0, https://github.com/rcastelo/GSVA)^55^ was utilized to apply the MSigDB gene sets (h.all.v2023.1.Hs.symbols.gmt) to TCGA-LIHC dataset. We sought to assess the functional enrichment variations between the LIHC and control groups. The criteria for selecting significantly enriched gene sets in the GSVA included p.adj < 0.05 and the top 10 positive or negative logFC values.

### Analysis of SMs and CNVs

The somatic mutation analysis for LIHC samples from TCGA-LIHC dataset used “Masked Somatic Mutation” data from TCGA website, preprocessed with VarScan software. The SM landscape was visualized using the R package maftools (Version 2.18.0, https://github.com/PoisonAlien/maftools)^56^. TCGA-LIHC dataset’s “Masked Copy Number Segment” data from TCGA website was utilized to analyze CNVs in LIHC samples. The CNV segments were analyzed using GISTIC2.0^57^ with default parameter settings after downloading and processing.

### Development of a prognostic risk model for LIHC

We utilized the R package survival (Version 3.5-7, https://github.com/therneau/survival)^58^ to conduct univariate Cox regression analysis on clinical data, aiming to create a prognostic risk model for LIHC using TCGA-LIHC dataset. We evaluated the prognostic impact of ICDR&CSRDEGs to determine their independence as prognostic factors. Genes associated with the prognostic risk model were identified through multivariate Cox regression analysis of variables with a p-value < 0.10 from univariate Cox regression. A forest plot depicted the expression levels of the model genes using univariate and multivariate Cox regression analyses. The risk score was derived from multivariate Cox regression analysis coefficients using the following formula:

$${\text{risk}}Score = \mathop \sum \limits_{i} Coefficient\left( {gene_{i} } \right)*mRNA\;Expression\left( {gene_{i} } \right).$$

### Analysis of the prognostic risk model for LIHC

LIHC samples were categorized into high- and low-risk groups using the median risk score from the prognostic model. Cox regression analyses, both univariate and multivariate, were conducted using the risk score alongside clinical variables including age, sex, and pathologic stage. Variables with a p-value less than 0.10 from the univariate analysis were subsequently evaluated using multivariate Cox regression. A forest plot was employed to illustrate the impact of the risk score and clinical variables from both analyses. A nomogram^59^, created with the R package rms (Version 6.7-1, https://github.com/harrelfe/rms) and derived from multivariate Cox regression analysis, visually represents the relationships between independent variables using distinct line segments, highlighting the association between the risk score and clinical data. A Kaplan–Meier curve analysis^60^ was conducted to assess patient survival times, examining the relationship between survival outcomes and influencing factors, with the curve derived from the risk score evaluating patient survival time.

The time-dependent ROC^61^ curve is a graphical tool for assessing model performance and selecting optimal models or thresholds. The timeROC (Version 0.4, https://cran.r-project.org/web/packages/timeROC/index.html)^62^ R package was utilized to create the curve and compute the AUC for evaluating the prognostic accuracy of the risk score. AUC values, ranging from 0.5 to 1, signify improved diagnostic performance with high scores. An AUC greater than 0.5 indicates event promotion, with values between 0.5 and 0.7 reflecting low accuracy, 0.7 and 0.9 indicating moderate accuracy, and values above 0.9 representing high accuracy. Calibration curves were used to compare the observed outcomes with the model predictions and assess the predictive accuracy and discrimination ability of the model. A calibration analysis was conducted to illustrate the comparison. The model’s clinical utility was assessed through DCA^63^ using the ggDCA (Version 1.1, https://cran.r-project.org/package=ggDCA)^64^ R package. The DCA diagram offers insights into the model’s accuracy and discrimination in predicting LIHC outcomes.

### Immune-cell infiltration analysis

The ssGSEA^65^ evaluates the relative abundance of immune-cell infiltrates, identifying immune cells such as activated CD8^+^ T cells, activated dendritic cells, gamma-delta T cells, natural killer cells, and Tregs. Enrichment scores from ssGSEA indicated the immune-cell infiltration levels in the samples. Samples with a p-value under 0.05 were utilized to construct the immune-cell infiltration matrix. The R package ggplot2 was used to generate plots comparing groups in the ssGSEA, highlighting differential expression between LIHC and control samples. Using the R package pheatmap (Version 1.0.12, https://cran.r-project.org/web/packages/pheatmap/index.html), heatmaps were created to illustrate the correlation between immune cells and the association of prognostic risk model-related genes with ssGSEA immune cells in both LIHC and control samples. Correlation coefficients (r-values) were categorized as follows: below 0.3 indicates weak or no correlation, 0.3 to 0.5 signifies weak correlation, 0.5 to 0.8 represents moderate correlation, and above 0.8 denotes strong correlation.

### Analysis of differential gene expression in prognostic risk models

We evaluated the expression differences of prognostic risk model-related genes between high- and low-risk groups in TCGA-LIHC dataset and LIHC samples by calculating risk scores for the combined datasets. The scores were calculated using risk coefficients from multivariate Cox regression analysis combined with the expression levels of the model genes. Samples were divided into high- and low-risk groups using the median risk score derived from the prognostic model formula.

$${\text{risk}}Score = \mathop \sum \limits_{i} Coefficient\left( {gene_{i} } \right)*mRNA\;Expression\left( {gene_{i} } \right)$$

Comparison maps were generated for high- and low-risk groups using TCGA-LIHC and combined datasets, emphasizing gene expression levels associated with the prognostic risk model. We created group comparison maps to analyze differences in model gene expression between TCGA-LIHC dataset and the combined datasets for both LIHC and control samples.

### Statistical analysis

We employed R software (version 4.3.1) for data processing and analysis. Continuous variables are expressed as mean ± standard deviation. Group comparisons were conducted using the Wilcoxon rank-sum test, while Spearman’s correlation analysis evaluated molecular correlations, with statistical significance defined as *p* < 0.05.

## Data Availability

Data are available in a public, open access repository. Data are available on reasonable request. All data relevant to the study are included in the article or uploaded as supplemental information. The datasets (GEO data) and (TCGA LIHC data) for this study can be found in the GEO (https://www.ncbi.nlm.nih.gov/) and TCGA (https://www.cancer.gov/about-nci/organization/ccg/research/structural-genomics/tcga).
